# Clinical outcome in Giant cell tumor of cervico-thoracic spine: Our experience with three cases

**DOI:** 10.1016/j.ijscr.2020.05.033

**Published:** 2020-05-29

**Authors:** Jagdeep Singh, Raj Bahadur, Sorabh Garg, Karan Rajpal, Karan Chopra

**Affiliations:** Department of Orthopaedics, Guru Gobind Singh Medical College and Hospital, Faridkot, India

**Keywords:** Biopsy, Excision, Giant cell tumor of the spine, Radiotherapy

## Abstract

**Background:**

Giant cell tumor (GCT) of the spine is uncommon but most aggressive benign tumor of the spine with unpredictable outcome. The purpose of this study was to report on a surgical treatment for the cases of GCT (C2, T4 and C7-T1). The spine is not a common site for a Benign GCT, with a 2.5% incidence in the sacrum and 2.9% in the vertebrae above the sacrum. In the cervico-thoracic spine, the incidence is extremely low and has been reported very less in the literature.

**Material and methods:**

This study was conducted on 3 cases of GCT of the spine and evaluated the outcome of different treatment modalities retrospectively. All the cases were treated with intralesional surgical resection but only one developed recurrence.

**Results:**

Cord compression and neurological deficits of varying grades was observed in all the cases. All patients also presented with clinical as well as radiological instability. Overall results were satisfactory, as all patients were symptom-free postoperatively. One out of the three cases had tumour recurrence and needed repeat intervention.

**Conclusion:**

Giant cell tumour of cervico-thoracic spine is a rare entity and should be managed Surgically with en bloc/extralesional resection but due to risk of surrounding neurovascular structures damage they are managed by marginal resection therefore since total resection is not possible there are high chances of recurrences, Hence require close monitoring and follow up for early diagnosis and appropriate management.

## Highlights

• Giant cell tumor (GCT) of the spine is uncommon but most aggressive benign tumor of the spine with unpredictable outcome. The purpose of this study was to report on a surgical treatment for the cases of GCT (C2, D4 and C7-D1). In the cervico-dorsal spine, the incidence is extremely low and has been reported very less in the literature.
• Local recurrence is high when total resection is not achieved, especially when the tumor extends around the neural elements or adjacent vascular structures. In the upper cervical spine, the recurrence rate is slightly higher because complete excision is not always feasible.
• Giant cell tumour of cervico-dorsal spine is a rare entity and should be managed Surgically with en bloc/extralesional resection but due to risk of surrounding neurovascular structures damage they are managed by marginal resection.
• Also radiotherapy plays a pivotal role to prevent recurrence in cases where extralesional/en bloc excision is not feasible if used in controlled dosage.

## 1 Introduction

Historically, GCT has been referred to by numerous terms, including myeloid sarcoma, tumor of myeloplaxus, osteoblastoclastoma, and osteoclastoma [[1], [2], [3], [4]]. It was first described by Sir Astley Cooper [5] in 1818; GCT is a relatively common skeletal benign and solitary tumor, accounting for 4%–9.5% of all primary osseous neoplasms and 18%–23% of benign bone neoplasms. However, multiple lesions have been described (although they are rarely associated with Paget disease), and 5%–10% of lesions may be malignant [[6], [7], [8], [9], [10], [11]].

Giant cell tumor (GCT) of bone is a rare neoplasm that accounts for approximately 5% of all primary bone tumors in adults. GCT most frequently occurs at the end of long bones, and the sacrum is the fourth most common site, accounting for between 1.7–8.2% of cases. Giant cell tumor also occurs in the mobile spine, but this location accounts for only 2–4% of cases [12]. In a review of literature done by Shankman et al. in 1988, 2.7% of GCTs were located in the spine [13]. Most of these benign neoplasms occur at sacrum followed by thoracic and cervical spine in a descending manner [14]. It occurs in the age group of 20–45 and sex incidence is equal [15].

Common symptoms of patients with spinal GCT include back pain, neurological deficit due to compression of spinal cord, bladder and bowel dysfunction, and structural deformity of the spine. Radiologically, spinal GCTs also present as expansile lytic lesion that most often involves the vertebral body [16] and soft-tissue involvement may be present. Additional imaging modalities including bone scintigraphy, computed tomography (CT), and magnetic resonance (MR) imaging are frequently used to evaluate these lesions for staging purposes and precise location.

Various modalities of treatment used for spinal GCTs are surgery, radiotherapy, embolization, cryosurgery, cementation, and chemical adjuvant like phenol or liquid nitrogen. Total *en bloc* surgical excision is the treatment of choice in long bones as well as spine. The treatment of long bones GCT is curettage, sclerotherapy, and filling of the defect with bone cement [17]. This is not always possible in the spine due to the unacceptable risk of permanent neurological deficit [18].

We are presenting a case series of THREE cases of GCT of cervico-thoracic spine and sharing our experience regarding their clinical outcome.

## 2 Material and methods

We retrospectively analyzed cases of spinal GCT that had been operated between 2015–2018. From the approximately 300 spinal surgeries done during this period 35 were reported to have tumor and only three were for GCT and formed our study. All the data was extracted from hospital records, including preoperative and sequential postoperative clinical findings, radiological details and pictures of the status at last follow-up. Patient was assessed by plain radiographs and CT-Scans for recurrence of tumour, instability and the status of fusion and spinal implants. There were one male and two females in our group of three patients. Age ranged from 22 to 30.

All the Three patients Presented as fresh cases and only one case had a recurrence after their first surgery with us. Two patients presented with cord compression and neurological deficit while one just had associated pain symptoms. All patients had clinical as well as radiological evidence of spinal instability judged on the basis of factors such as Mechanical pain, Location of lesion, Bone lesion type, Radiographic spinal alignment, Vertebral body collapse, Postero-lateral involvement used for calculating Spinal Instability Neoplastic score (SINS) [19]. All cases showed higher SINS score.

**Fig. 1:**
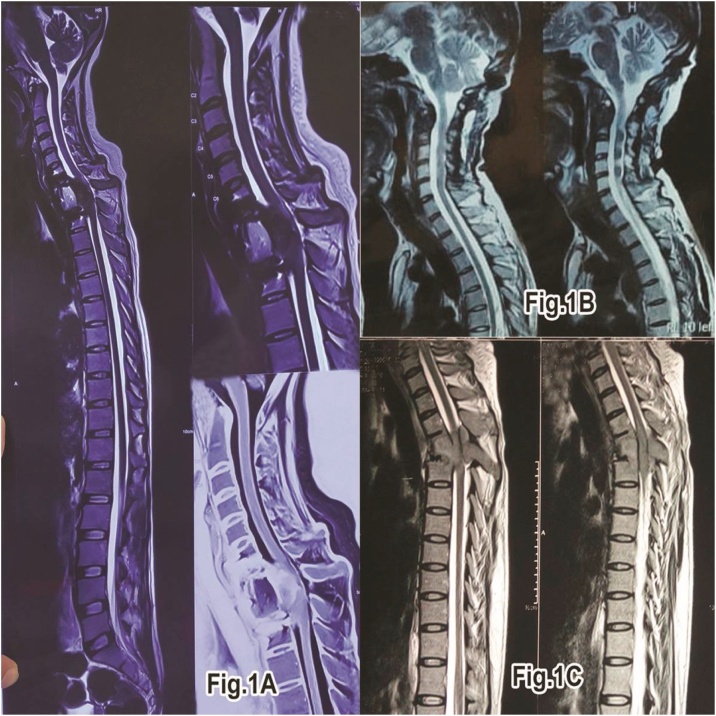
Pre-operative MRI images saggital section showing the ocation of GCT. A:MRI images showing GCT at C7-D1 region. B:MRI images showing GCT at C2 level. C:MRI images showing GCT at D4 level.

One of our pt had GCT in cervical spine (C2 level), One in Thoracic spine (T4 level) and third in cervico-thoracic junction (C7-T1 level) [Fig. 1]. All the cases were aptly followed up in view of tumour recurrence and instability but only one case reported recurrence out of the three after being normal for a period of 6–8 months and also thereafter after second intervention is free from symptoms upto the last follow up (Table 1).

**Table 1:** Summary of data of three patients.

S. no.	Level of GCT	Sex	Age (year)	Surgical intervention	recurrence	Additional treatment	2^nd^ surgical intervention	Follow up (years)	Neurological Deficite
1	C2	Male	24	Anterior excision along with posterior fusion and stabilization	Not reported	Radiotherapy	Not required	2yrs and asymptomatic	Neck Pain with no radiating pain and no Neurological Deficiet
2	C7-T1	Female	24	Anterior excision with corpectomy C7-T1	Not reported	Not reported	Not required	2yrs and asymptomatic	Neck pain with complete paraplegia and planter extensor
3	T4	Female	30	Anterior thoracotomy with tumor excision and rib graft fixation	Reported	Radiotherapy	Posterior decompression and fixation from T3 to T5	2yrs and asymtomatic	Complete paraplegia with bladder bowel involvement and sensory deficiet below T4-T5 with planter extensor

**Fig. 2:**
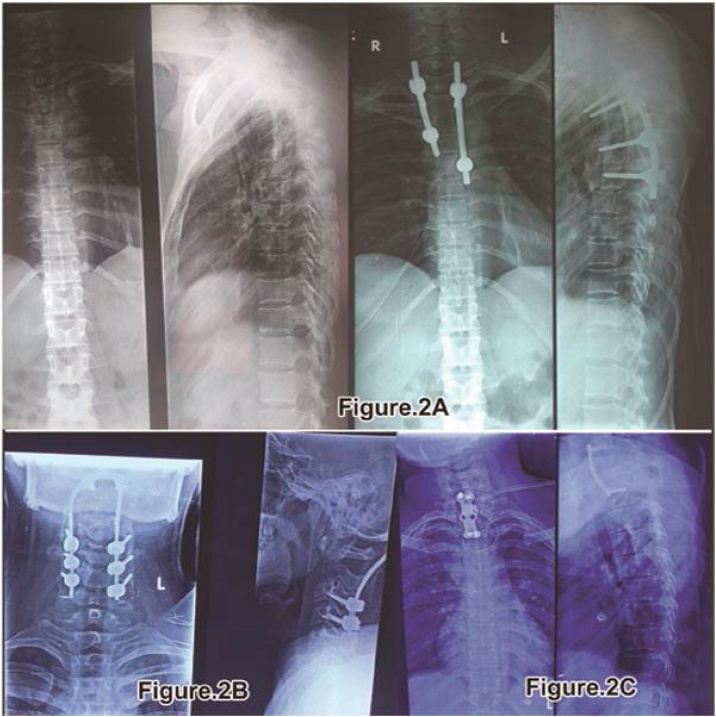
Post-operative xray images showing the surgical intervention the patient underwent. A:Post-operative xray showing rib graft in situ and posterior fixation done after recurrence. B:Post-operative xray showing Lateral mass screw fixation at C2 level. C:Post-operative xray showing long cervical plate in situ after corpectomy C7-D1.

**Fig. 3:**
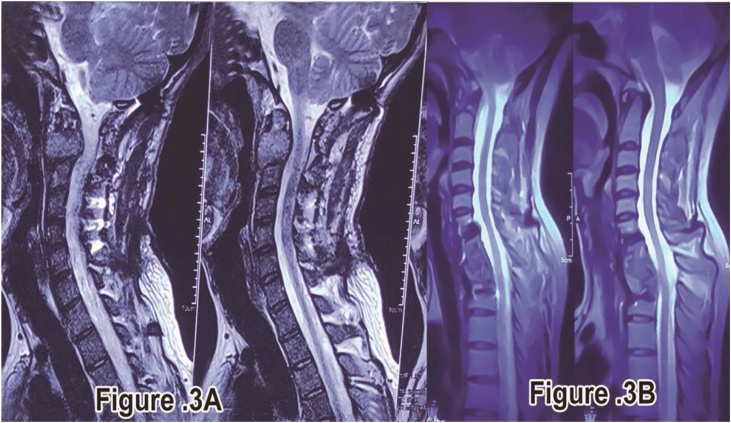
Post-operative MRI image on Long term follow up. A:MRI image showing good alignment and no cord compression at C2 level. B:MRI image showing good alignment and no cord compression at C7-D1 level.

In our first case study of 24-year-old male who presented to author institute Hospital OPD with the complaint of pain in the neck of 2 months duration. Onset was insidious and the pain was worsening. Pain was located in the mid cervical-spine radiating bilaterally to the upper limbs. Neck movements were grossly restricted and painful along with difficulty in daily activity. NO history of loss of appetite/weight with NO history of fever. No neurological deficit was present in the upper and lower limbs. Marked tenderness was present in the mid cervical region. Patient was advised plain radiograph cervical spine. Plain radiograph showed destruction C2 vertebrae body. Patient was advised MRI [Fig. 1] and corresponding reporting was done which showed expansile enhancing lesion involving C2 vertebrae with foci of T1 hyperintensity and blooming on GRE s/o haemorrhage and extension into prevertebral space suspected GCT. Patient was planned for biopsy after complete Pre-Anesthetic check-up and informed consent and excision biopsy was done using anterior approach. Excised material was sent for Histopathological examination. Patient was immobilized using crutch field tongs (CFT) till then. After 2 weeks, Report showing tumour composed of sheets of mononuclear stromal cells and uniformly distributed giant cells throughout the lesion confirmed GCT then Posterior fusion and stabilization surgery was done by posterior fixation from (C3, C4, and C5) to occiput [Fig. 2] and washed with hydrogen peroxide(H2O2) and Normal saline(NS) under General Anesthesia (GA) was done. Postoperatively the patient improved, allowed to do sitting and walking and discharged under stable conditions with stitch out at 2 weeks. The patient underwent 2–3 cycles of radiation therapy for the same. The patient is advised follow-up and physiotherapy for one year. On regular follow-up after 1 year MRI [Fig. 3]. Showed good alignment and no sign of recurrence of GCT. Overall the patient is free of pain and made a good recovery. Patient will be followed-up periodically due to high risk of recurrence of these tumors following intra-capsular excision.

In one of the case of 24 year old female with complete paraplegia from one month with complains of pain in neck region from last three months. Power in the lower limbs was grade – 0/5 and in the upper limbs was grade – 4/5 with Plantor-Extensor. Patient was advised Plain radiograph of the cervico-thoracic spine which showed compression at C7-T1 with decrease in height of C7 and T1. Patient MRI showed “Areas of altered signal intensity involving C7 and T1 vertebrae and C7-T1 intervertebral disc involvement with Anterior wedging, Focal Kyphosis at C7-T1 level with epidural mass causing thecal sac indentation and cord compression at these levels with cord edema at C6 level with pre and paravertebral abscess at C7-T1 level”, compressing/encasing respective exiting Nerve roots [Fig. 1]. Patient was planned for Excision and biopsy through anterior approach along with corpectomy C7-T1. CT scan advised to mark the level of erosion at the vertebrae at C7-T1. Complete excision of C7-T1 done and washed with hydrogen peroxide (H2O2) and Normal saline (NS) along with Fusion and Cervical plate fixation [Fig. 2]. Complete cord compression released, specimen excised and sent for histopathological examination which also proved GCT of vertebrae. Stitches removed and patient was discharged after two weeks. Patient followed up in OPD after one month with no signs of improvement and then was managed conservatively along with Passive physiotherapy to continue. Patient was referred to radiotherapy department for further management but patient refused for the same. Patient followed up in OPD after five months with improvement in sensations in lower limbs and power grade-2/5, Plantor Extensor. Patient was advised corresponding appropriate physiotherapy management with neuro-muscular stimulants. Patient followed up at seven months with grade-3/5 power and Plantor-Extensor with increased sensations over bilateral lower limbs. Patient followed up in OPD at one year with grade-5/5 power and Plantar-Flexor with sensations- WNL and patient was advised Plain radiograph and MRI which showed “Post operative status at C7-T2 with anterior cervical plate in place and abnormal residual signal in central spinal canal on left side with mild compression and displacement of spinal cord with focal myelomalacic change at C6-C7 level” [Fig. 3]. Patient was advised regular follow up three monthly and till now is asymptomatic and walking and doing daily routine activities. No neurological complains and deficit

In other case of 30 year old female presented in OPD with complete paraplegic from one month with Bladder Bowel involvement along with sensory deficit below T4-T5 with Planter extensor reflex. History of loss of appetite present but NO history of fever/loss of weight.ESR levels were 45 mm/hr. Patient was advised Plain radiograph of the thoracic spine which showed collapse of T4 with decrease in height of the same. Patient on MRI showed Compressive Myelopathy with collapse at T4. MRI [Fig. 1] reporting showed “collapse with compression of cord along with soft tissue in epidural and paravertebral space at the level of T4 vertebrae”. As the patient presented with complete paraplegia and baldder/bowel involvement direct Anterior Decompression was planned. Complete excision of the tumor at the level of T4 done as much as possible and washed with hydrogen peroxide (H2O2) and Normal saline (NS) and decompression of the cord was done with using rib graft for fixation between T3-T5. Post-operatively patient was on self induced catheterization due to bladder bowel involvement. Histopathological examination was suggestive of GCT. Stitches removed and Patient was discharged after 2 weeks. Neurologically patient improved after 3–4 weeks with improvement of power at knee/Ankle/hip up to grade-3/5. Patient underwent 1–2 cycles of additional radiotherapy treatment. After 3–4 months patient regained bladder sensation and control along with improvement in power as well up to grade 4/5 and able to bear weight over lower limbs and do walking with walker. Patient was asymptomatic for 6–8 months then patient come to hospital with weakness of bilateral lower limb with Bladder Bowel involvement with Power grade-3/5 and Planter Extensor. Again fresh Plain radiograph and MRI of the patient was conducted which showed recurrence at the level of T4 with posterior spine elements involvement.MRI reporting showed Recurrence/Residual changes of altered signal intensity at T4 level with cord compression. Patient was again planned for surgery from posterior approach for T4. Posterior decompression and fixation was done from T3 to T5 [Fig. 2]. Patient was taken due care and was discharged after two weeks on self induced catheterization. After three months of follow-up, patient showed sign of improvement with Power upto grade-4/5 and was planned for radiotherapy (EBRT) and chemotherapy. Since then patient is on regular follow-up from last 2years and is asymptomatic and also walking with No neurological complaints and deficit.

## 3 Results

In Our 4 interventions we achieved goals of spinal decompression and stabilization. All three returned to neurologically stable state with adequate bladder bowel control at the last follow up. The patient with recurrence showed signs of improvement of power upto grade 4/5 with bladder bowel continence.

We had no major complications, though one patient in a long term follow-up presented with recurrence after 6–8 months of normal interval. Posterior decompression was planned in view of MRI showing involvement of posterior spinal elements which underwent successfully and is free from symptoms on further follow-up till date.

Out of the three cases two underwent postoperative radiotherapy for which doses varied depending on type of lesion and previos radiotherapy.

All the three patients on subsequent follow ups after getting plain radiographs and MRI done postoperatively at long term follow-up showed Good alignment and no recurrence of GCT [Fig. 3]. Overall patients are free of pain(asymptomatic) and made a good recovery in terms of walking and no neurological deficit. Patients are still under regular follow up periodically due to high risk of recurrence of these tumors following intra-capsular excision.

## 4 Discussion

The spine is not a common site for a Benign GCT [23], with a 2.5% incidence in the sacrum and 2.9% in the vertebrae above the sacrum [24]. In the cervical spine, the incidence is extremely low and has been reported to be less than 1% in the literature [25,26]. GCT is commonly seen in the 20–45 years age group [27] similar to the cases studied in this study. Pain in the area of the tumor is the most common symptom for the patients; however swelling and limited range of motion in the affected joint is noticed [28].

Extensive previous studies in the literature show paucity of data regarding incidence and management of GCT cervico-thoracic spine. GCT of sacrum and lumbar spine has been well reported [12,29]. A study conducted by Shekhar Y Bhojraj et al. retrospectively on 200 cases of spine surgeries done from 1993 to 2006 had only 6 cases of GCT spine and out of that only one was of GCT Cervical spine [20]. Similarly a study done by Chritopher martin et al. on 23 cases of GCT spine only 5 cases of GCT cervical spine were reported [30]. It states GCT of Cervical spine is of an uncommon Nature.

The gross appearance of a GCTB is described as having a vascular, friable tissue with a surface that is reddish brown, yellowish brown or yellowish gray [31]. Differentiation between GCTBs and aneurysmal bone cysts (ABCs) may be challenging. A young age suggests ABCs, whereas an older age suggests GCTB [32]. Lesions above the sacrum were once believed to be ABCs until proven otherwise [33].

Spinal GCTs are usually expansile lesion with bone destruction that affects the vertebral body and can cross disk spaces and extend into the posterior elements, but involvement in the posterior elements was observed with other spinal bone tumors, as aneurismal bone cyst and osteoid osteoma or osteoblastoma [34]. Posterior elements involvement was reported in one of our cases with recurrence of symptoms and neurological deficit and the same was treated with revision surgery using posterior approach and is till follow up is asymptomatic. On MRI, GCTB may show heterogeneous or homogeneous signal intensity on T1-weighted images with possible areas of high-signal intensity caused by recent hemorrhage. The solid areas of the tumor have heterogeneous low to intermediate signal intensity on T2-weighted images. In more than 50% of cases, areas of low signal intensity may be exaggerated on T2-weighted, spin-echo images due to the presence of hemosiderin [35].

Local recurrence is high when total resection is not achieved, especially when the tumor extends around the neural elements or adjacent vascular structures. The overall recurrence rate in the spine is between 25 and 45% [36]. In the upper cervical spine, the recurrence rate is slightly higher because complete excision is not always feasible.

In the spine, the initial surgery of GCT should be as aggressive as possible within neurological preservation and spinal stability. In case of partial removing of tumors, the radiation therapy is a useful added treatment with less risk of recurrence even though it gives a chance of malignant transformation [20]. Its high rate of recurrence i.e. 2%–25% of cases, means that there is no consensus about its management. Periodic CT and MRI are excellent tools to identify the recurrent lesion and plan out the necessary treatment. Patient advised regular follow up as recurrence is high and one should be vigilant enough to monitor signs, Corresponding required investigation (plain radiograph/ct/mri) should be repeated at regular intervals for early detection and diagnosis. A Adjuvant radiotherapy should be reserved for incomplete tumor excision and local recurrence due to risk of myelitis and bone graft complications [30].

Similar to our inference extensive study of the literature showed Giant cell tumors of the spine including sacrum should be managed with en bloc resections whenever possible as this provides the greatest chance for cure but When the risk of post-operative neurologic deficit after en bloc excision is high they should undergo intra lesional marginal resection followed by controlled local radiotherapy [12,37].

The recommended follow up for spinal GCTs in view of high recurrence rate is three monthly for initial two years followed by six monthly for three years and then yearly for 5 years making it completely around 10 years along with CT Scans and MRI at corresponding follow ups. The previous reported studies in the past had a mean follow up of 5 years [[20], [21], [22]]. Similar trends were followed in our study for periodic follow up and were assessed and evaluated for any recurrence of symptoms. CT scans/MRI radiography was done at yearly interval in Non symptomatic patients. The patients are on regular follow-up from last 5 years and are asymptomatic with No neurological complaints and deficit.

Usually, some microscopic tumor tissue is expected to stay behind, therefore postoperative radiotherapy is recommended. Though earlier literature seemed to suggest that irradiation converts benign GCTs to malignant ones, this is no longer true with modern radiotherapy techniques, especially by keeping the total radiation dose under 50 Gy [37].This Research work has been reported in line with the PROCESS criteria [38].

## 5 Conclusion

Giant Cell Tumour of the cervico-thoracic spine is a rare entity which has not been previously extensively reported. Early diagnosis and proper surgical excision using extralesional/en bloc resection is required but due to the fear of injury to the extremely close neurovascular structures Marginal resection is done which leads to higher chances of recurrence as the total resection is not feasible. A close follow-up and monitoring for signs and symptoms with periodic imaging are excellent tools to identify the recurrent lesion and plan out the necessary treatment. Also radiotherapy plays a pivotal role to prevent recurrence in cases where extralesional/en bloc excision is not feasible if used in controlled dosage.

## Declaration of Competing Interest

The authors declare that they have no conflict of interest.

## Sources of funding

There was no sources of funding for our research.

## Ethical approval

Since we are not reporting the first case of this type in the literature, hence no approval was taken from the relevant ethics committee but written informed consent was taken from the patient to publish his details and X-ray photographs.

## Consent

Written informed consent was obtained from the patients for publication of this case report and accompanying images. A copy of the written consent is available for review by the Editor-in-Chief of this journal on request.

## Author contribution

SG and JS were involved in Conceptualization and Data curation. JS, SG and KR did Formal analysis and Investigation. SG, RB and JS formulated Methodology. JS and KC did project administration. SG and KR developed Resources and Software. RB and JS Supervised. SG, JS and KR Validated and Visualized. SG involved in Writing - original draft. JS helped in Writing - review & editing.

## Registration of research studies

NA.

## Guarantor

Dr. Sorabh Garg.

## Provenance and peer review

Not commissioned, externally peer-reviewed.
